# Conformational Analysis of the Oligosaccharides Related to Side Chains of Holothurian Fucosylated Chondroitin Sulfates

**DOI:** 10.3390/md13020936

**Published:** 2015-02-12

**Authors:** Alexey G. Gerbst, Andrey S. Dmitrenok, Nadezhda E. Ustyuzhanina, Nikolay E. Nifantiev

**Affiliations:** N.D. Zelinsky Institute of Organic Chemistry, Russian Academy of Sciences, Leninsky prospect 47, 119991 Moscow B-334, Russia; E-Mail: dmt@ioc.ac.ru

**Keywords:** fucosylated chondroitin sulfate, oligosaccharides, conformational analysis, glycosidic linkage, Karplus equation, long range C–H coupling constants

## Abstract

Anionic polysaccharides fucosylated chondroitin sulfates (FCS) from holothurian species were shown to affect various biological processes, such as metastasis, angiogenesis, clot formation, thrombosis, inflammation, and some others. To understand the mechanism of FCSs action, knowledge about their spatial arrangement is required. We have started the systematic synthesis, conformational analysis, and study of biological activity of the oligosaccharides related to various fragments of these types of natural polysaccharides. In this communication, five molecules representing distinct structural fragments of chondroitin sulfate have been studied by means of molecular modeling and NMR. These are three disaccharides and two trisaccharides containing fucose and glucuronic acid residues with one sulfate group per each fucose residue or without it. Long-range C–H coupling constants were used for the verification of the theoretical models. The presence of two conformers for both linkage types was revealed. For the Fuc–GlA linkage, the dominant conformer was the same as described previously in a literature as the molecular dynamics (MD) average in a dodechasaccharide FCS fragment representing the backbone chain of the polysaccharide including GalNAc residues. This shows that the studied oligosaccharides, in addition to larger ones, may be considered as reliable models for Quantitative Structure-Activity Relationship (QSAR) studies to reveal pharmacophore fragments of FCS.

## 1. Introduction

Anionic polysaccharides of a different nature were shown to affect various biological processes, such as metastasis, angiogenesis, clot formation, thrombosis, inflammation, bacterial and viral adhesion, and some others [1,2,3,4]. In some cases, exogenous polysaccharides act *via* the mechanism of competitive inhibition of the natural ligand binding to the target protein. Thus, well known glycosaminoglycan heparin was found to reduce inflammation and metastasis in a way of inhibition of P- and L-selectins binding to their cellular ligands [4,5]. In other cases, carbohydrates could potentiate interaction of two proteins with the formation of a ternary complex, which can be illustrated by thrombin-antithrombin III-heparin complex formation playing the key role in inhibition of blood coagulation [6,7]. However, side effects of heparin treatment, such as bleeding and thrombocytopenia, force the search for and development of alternative drugs. Among the compounds under investigation are the polysaccharides fucosylated chondroitin sulfates (FCSs) isolated from different holothurian species. These biopolymers demonstrated a wide range of biological activities including antimetastatic, anti-inflammatory, anticoagulant, antithrombotic, immunostimulatory, antibacterial, antiviral, and some others activities typical of heparin but with a lack of side effects [3,8,9,10,11,12,13].

**Figure 1:**
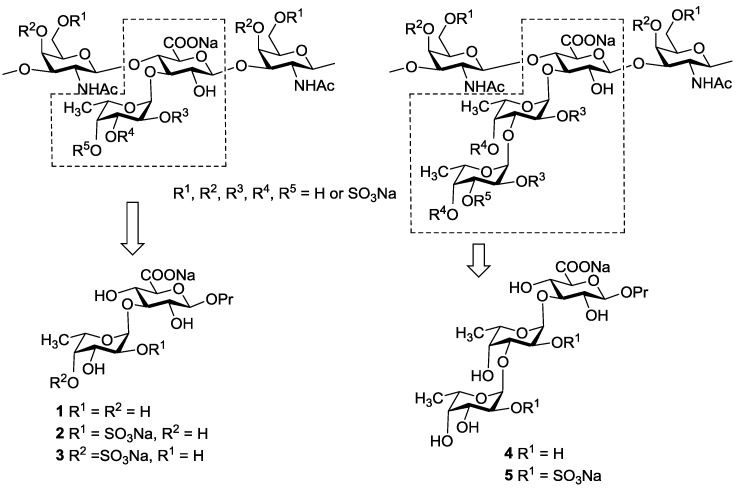
Branched fragments of fucosylated chondroitin sulfates and synthetic oligosaccharides related to the knots.

The backbone of known FCSs is built up of alternating →4)-linked β-d-glucuronic acid and →3)-linked *N*-acetyl β-d-galactosamine residues. Some of glucuronic acid units are substituted at *O*-3 by selectively *O*-sulfated fucosyl residues (Figure 1) [3,13]. The presence of side chains in FCS distinguishes them from mammalian chondroitin sulfates and was shown to be essential for biological properties of FCS [13,14]. The pattern of *O*-sulfation and the structure of branches vary accordingly to the type of holothurian species and are basically responsible for the level of biological effect of FCSs [3,13]. Fine structural characteristics of these polysaccharides also depend on the geographic range, season of harvesting, and the method of isolation [13,15]. In Figure 1 the known structures of the branched fragments of FCSs are presented.

It is known that the presence of sulfate groups in these polysaccharides is crucial for the observed biological activity [1,3,11,13,14]. However, influence of certain sulfation patterns on the activity is poorly understood. In our laboratory, we have started a complex investigation of oligosaccharides related to various fragments of FCS, including their systematic synthesis, conformational analysis, and study of biological action, particularly the role of sulfate groups.

To understand the mechanism of FCSs action, knowledge about the spatial arrangement of these carbohydrates is required. In this communication, five molecules representing simple structural fragments of chondroitin sulfate have been studied by means of molecular modeling and NMR. These are three disaccharides (Figure 1) and two trisaccharides containing fucose and glucuronic acid residues with a single sulfate group per each fucose residue. The synthesis and detailed characterization of these compounds are described in [16].

## 2. Results and Discussion

**Figure 2:**
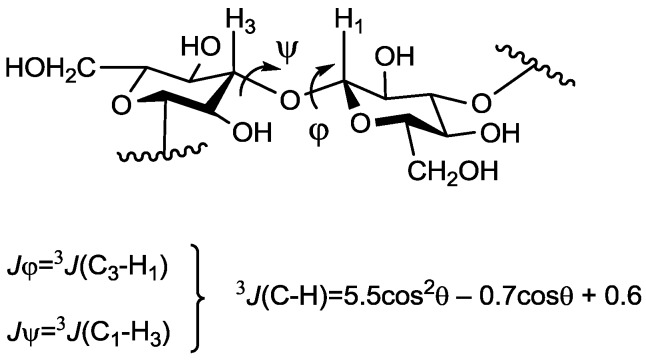
Torsional angles describing a glycosidic linkage and the Karplus equation [17]. Angles φ and ψ are defined as H_1_–C_1_–O–C_x_ and C_1_–O–C_x_–H_x_ correspondingly.

The conformation of the glycosidic linkage is usually described in terms of two torsional angles, φ and ψ. These are directly connected with values of long-range ^13^C–^1^H spin–spin coupling constants (SSCC, ^3^*J*_C–H_), which can be measured by various NMR techniques. The relationship between these parameters may be formalized by the Karplus type equation [17] (Figure 2), which opens the way towards the verification of the results of theoretical modeling by NMR methods.

**Figure 3:**
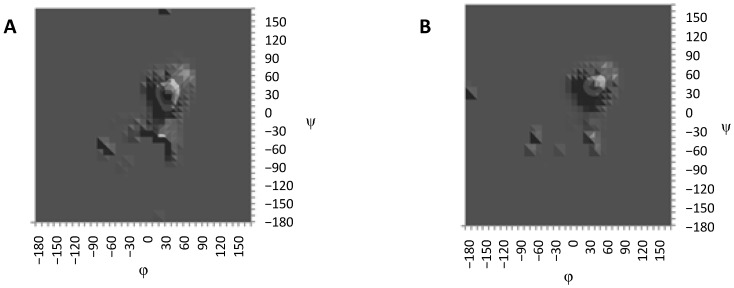
Sample confomational maps obtained by means of torsion scanning for Fuc–GlA (**A**) and Fuc–Fuc (**B**) linkages.

First, conformational maps were constructed for each oligosaccharide. A simple dihedral driving algorithm was used with a step of 10° for each torsion determining the conformation of the glycosidic linkage. Energies of the conformations in each step were plotted as conformational maps (Figure 3). In the modern conformational analysis of carbohydrates, such approach is considered as a very rough estimate of the potential energy surface. For each linkage in the studied compounds, only one populated area corresponding to the global minimum was revealed. Other minima had significantly higher energies and were scarcely populated. Thus, these results were not used for the modeling of the NMR parameters, and only two samples of the obtained maps are given in Figure 3.

The found minima were used as starting points for molecular dynamics (MD) calculations. These were carried out in three modes: first *in vacuo*, then with the account for solvation using the Solvent Accessible Surface Area (SASA) [18] continuum model, and with the explicit water molecules in a droplet. Such an approach was successfully used by us previously [19] during the conformational analysis of glucan fragments.

The general MD procedure included a 20,000 ps run for each starting structure with a snapshot being written at an interval of 2 ps, resulting in 10,000 snapshot structures in each trajectory. The constant temperature (298 K) protocol was used. The snapshot structures collected during MD simulations were used to calculate an ensemble of averaged SSCC values in order to compare them with the experimentally observed ones. The latter were measured employing J-HMBC technique (see the Experimental Section for details). Previously, the experimental error of thus measured ^3^*J*_C–H_ was found by us to have the value of 0.5 Hz [20].

*In vacuo* calculations quite expectedly produced results inconsistent with the experiment in all the cases (Table 1). An average difference between the experimental and calculated SSCC values was about 0.8 Hz. This can be attributed to the highly charged nature of the studied molecules and, obviously, a consideration of the solvation effects was required.

**Table 1:** Experimental and calculated with different models spin–spin coupling constants (SSCC) values for compounds **1**–**5**, Hz.

Compound Number	*J*φ (Fuc–GlA)	*J*ψ (Fuc–GlA)	*J*φ (Fuc–Fuc)	*J*ψ (Fuc–Fuc)
**1**	**3.8 (exp)**	**2.5 (exp)**	n.a.*	n.a.*
	4.0 (*in vacuo*)	3.8 (*in vacuo*)		
	3.4 (SASA)	2.7 (SASA)		
	3.2 (water)	2.5 (water)		
**2**	**2.7 (exp)**	**2.9 (exp)**	n.a.*	n.a.*
	4.0 (*in vacuo*)	3.7 (*in vacuo*)		
	3.6 (SASA)	2.3 (SASA)		
	3.2 (water)	2.4 (water)		
**3**	**3.0 (exp)**	**2.7 (exp)**	n.a.*	n.a.*
	4.0 (*in vacuo*)	3.8 (*in vacuo*)		
	3.3 (SASA)	2.7 (SASA)		
	3.2 (water)	2.6 (water)		
**4**	**3.6 (exp)**	**3.0 (exp)**	**2.8 (exp)**	**2.5 (exp)**
	4.0 (*in vacuo*)	3.8 (*in vacuo*)	3.5 (*in vacuo*)	3.2 (*in vacuo*)
	3.2 (SASA)	2.9 (SASA)	2.7 (SASA)	2.6 (SASA)
	3.3 (water)	3.4 (water)	2.5 (water)	2.5 (water)
**5**	**2.7 (exp)**	**2.5 (exp)**	**2.6 (exp)**	**2.9 (exp)**
	4.0 (*in vacuo*)	3.7 (*in vacuo*)	3.3 (*in vacuo*)	3.1 (*in vacuo*)
	3.2 (SASA)	2.4 (SASA)	3.2 (SASA)	3.4 (SASA)
	3.2(water)	2.4 (water)	2.8 (water)	2.6 (water)

* n.a.—Not applicable.

**Figure 4:**
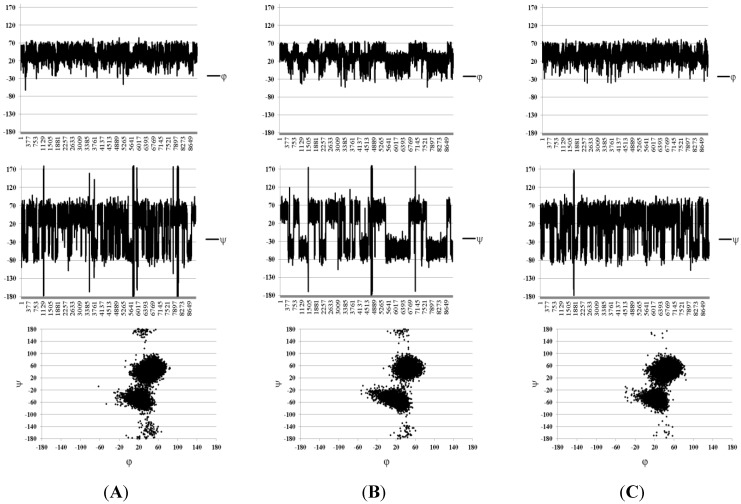
Molecular dynamics (MD) graphs and conformational plots obtained in the Solvent Accessible Surface Area (SASA) approximation for disaccharides **1**–**3** ordered from (**A**) to (**C**). Angles φ and ψ are defined as H_1_–C_1_–O–C_x_ and C_1_–O–C_x_–H_x_, respectively, for the sake of compatibility with the Karplus equation.

**Figure 5:**
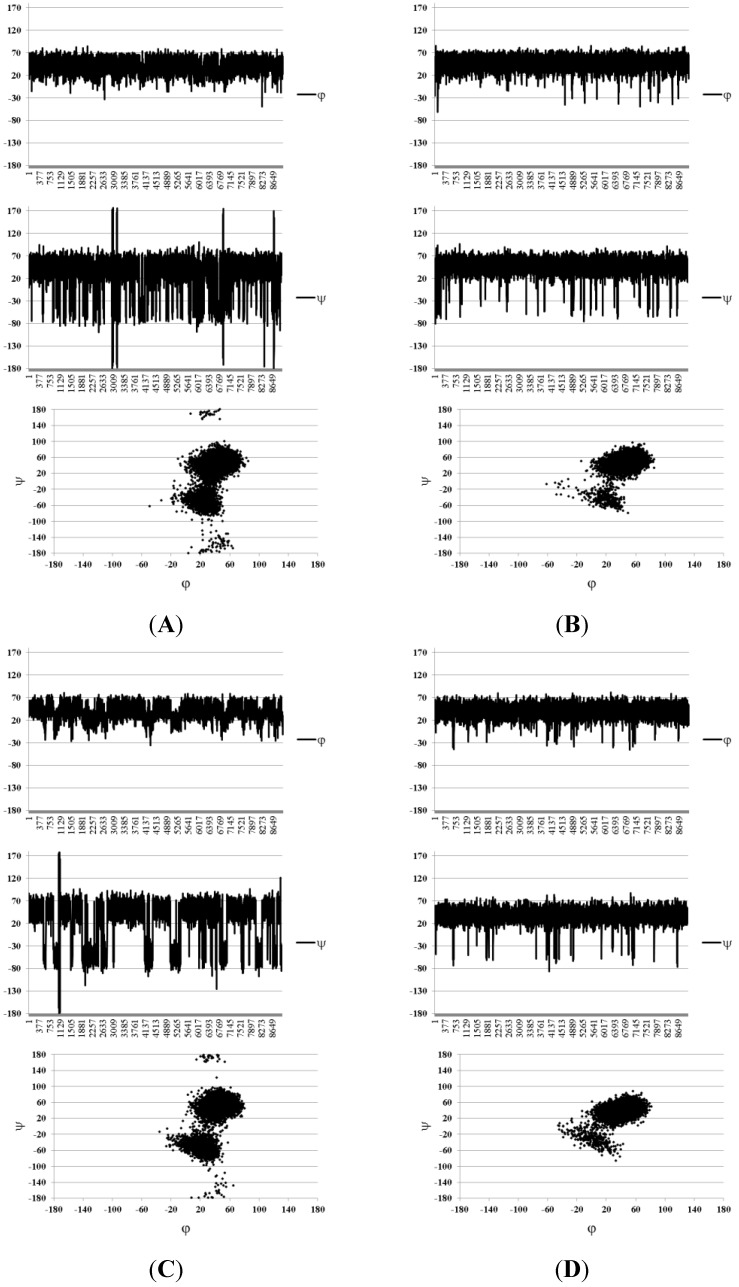
Conformational plots obtained in the SASA approximation for glycosidic linkages in structures **4** ((**A**), Fuc–GlA; (**B**), Fuc–Fuc) and **5** ((**C**), Fuc–GlA, (**D**), Fuc–Fuc). Angles φ and ψ are defined as H_1_–C_1_–O–C_x_ and C_1_–O–C_x_–H_x_, respectively, for the sake of compatibility with the Karplus equation.

First, the continuum solvation model SASA was tested and its use significantly improved the situation. As can be seen from Table 1, the calculated SSCC values became closer to those observed experimentally. The average deviation in this case was 0.34 Hz, which is within the experimental error. To identify the details of the conformational behavior of the studied molecules, graphs of angle change in time during MD and plots of the conformers obtained from MD simulations were constructed in coordinates φ/ψ (Figure 4 and Figure 5).

For two types of the glycosidic linkages (Fuc–Fuc and Fuc–GlcA) in these compounds, slightly different conformational distributions were observed. Both were characterized with the presence of conformers which are common among carbohydrates, with the torsional angles φ lying around 20°–40° (these are determined by the exo-anomeric effect), and ψ angles forming two clusters around +40° (conformer **I**) and −0° (conformer **II**).

In the case of Fuc–GlA linkages in all compounds, trace amounts of the inverted conformers were found, which had ψ torsions in the region close to ±170–180° (conformer **III**). Such conformers are sometimes encountered in the conformational distribution of carbohydrates but are usually poorly populated. In this case, their appearance may be explained by the relatively free rotation around the linkage Fuc–(1→3)–GlA due to the lack of axially oriented substituents in the glucuronic residue, contrary to the case of the linkage Fuc–(1→3)–Fuc. In general, these distributions resemble the conformational maps obtained by the dihedral scanning procedure (Figure 3). In this case, however, one can see that the conformers that previously appeared minor and insignificant (namely, **II** and **III**) now have become populated to a greater extent.

**Figure 6:**
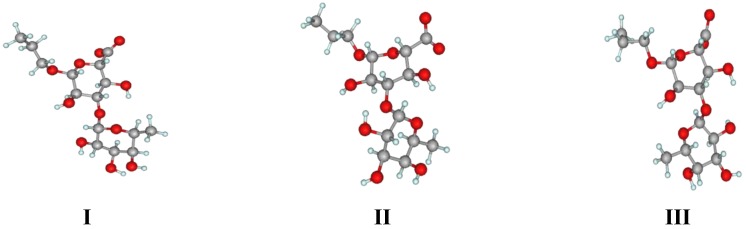
Principal conformers of the Fuc(1→3)GlA linkage.

Graphical representations of the detected conformers are given in Figure 6. Interestingly, conformer **I**, which, according to our results, seems to be predominant for the Fuc–GlcA linkage, has the same orientation of the monosaccharide residues as determined for this fragment in [11] during the MD analysis of two dodecasaccharides representing the backbone chain of the polysaccharide including GalNAc residues.

However, when the explicit water model was applied, the disappearance of the above mentioned inverted conformers was observed. It was accompanied also by a slight improvement of coincidence between the calculated and experimental SSCC values for all the studied compounds (Table 1).

**Figure 7:**
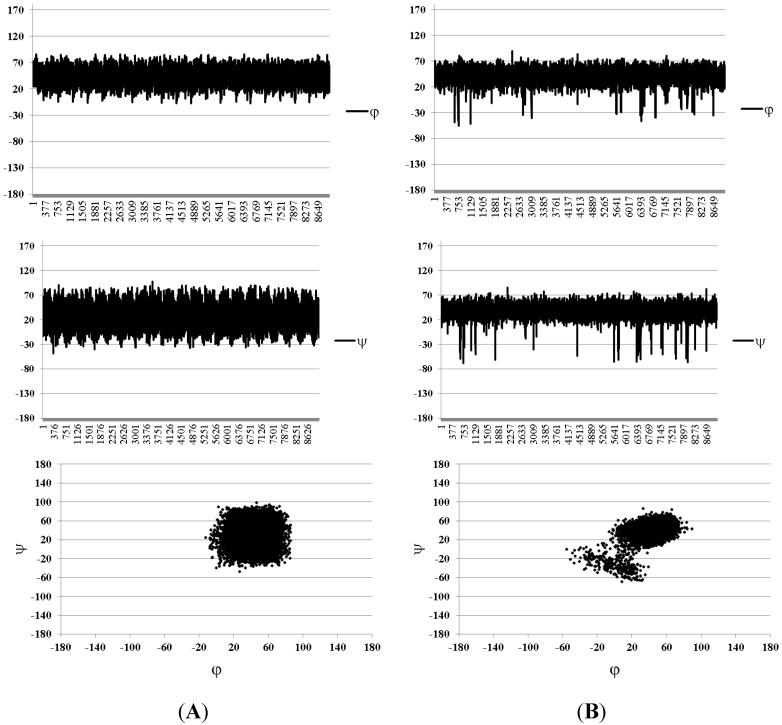
Torsional angles graph and conformational plots obtained using explicit water approximation for the fucosyl–glucuronide (**A**) and difucoside (**B**) fragments. Data are extracted from simulations of compound **4**. Angles φ and ψ are defined as H_1_–C_1_–O–C_x_ and C_1_–O–C_x_–H_x_ correspondingly for the sake of compatibility with the Karplus equation.

**Figure 8:**
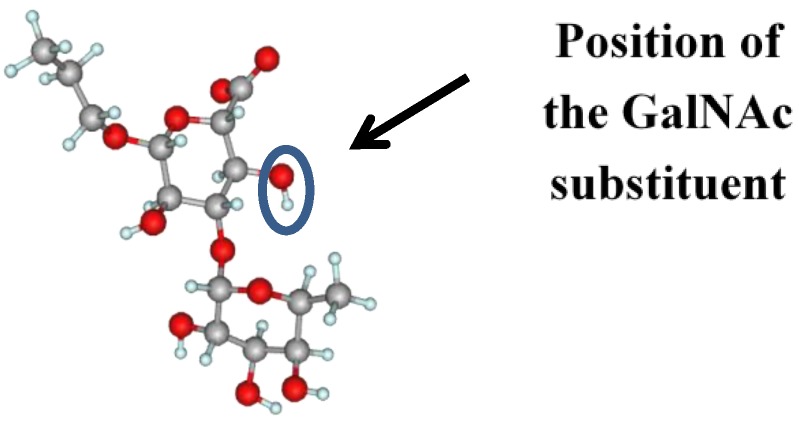
The dominant conformer of the Fuc(1→3)GlA linkage with the position of GalNAc residue introduction.

The sample MD graphs of the glycosidic torsions and the conformational plots obtained under these conditions for the Fuc–GlcA and difucoside fragments are shown in Figure 7. They are extracted from trajectories obtained during the simulations of trisaccharide **4**. It is noteworthy that during the studies of β–(1→3)–glucan related oligosaccharides [19] the same tendency for the inverted conformers was found. In fact, the dominating conformer for the Fuc–GlcA linkage was still that of type **I** (Figure 6), indicating that the conformation of the fucosyl–glucuronyl branch is essentially the same both in the presence and in the absence of the units of the backbone chain. The explanation of this fact can be achieved by closer examination of conformer **I**. The mutual orientation of fucose and glucuronic acid residue in it is such that the O-4 atom of the GlcA-unit is not hindered by the fucosyl substituent (Figure 8). Thus, it can be thought that the introduction of a galactosamine residue at this position should not change the conformation of the branch substantially.

**Figure 9:**
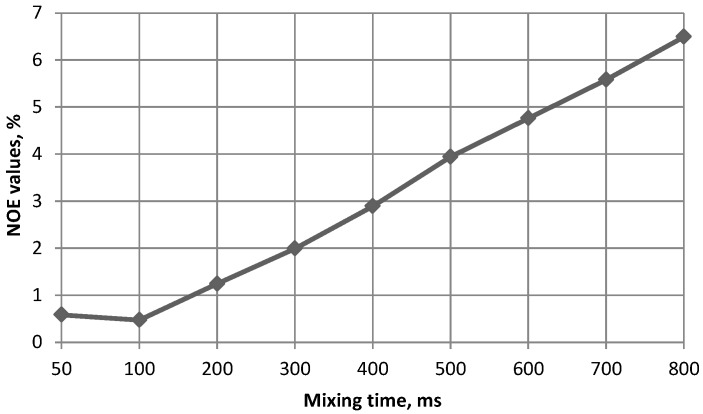
The NOE build-up graph at different mixing times for compound **4**. The measured NOE values are for H1(Fuc)/H3(GlA) interaction.

Additional evidence that actually no inverted conformers were present in the conformational distribution was achieved by means of NOESY spectroscopy. The mixing time used in these experiments was 500 ms. For the studied types of molecules in diluted solutions such mixing time gave reliably measurable NOE values and still was within the linear range of the NOE build-up. This fact was confirmed by conducting a series of 1D NOESY experiments using different mixing times on compound **1** (Figure 9). Slight non-linearity at 50–100 ms could be explained by the relatively small sensitivity in this range which increased the integration error.

The relative NOE values from MD simulations were calculated as the reciprocal ratio of the averaged sixth order of the corresponding interatomic distances. The NOE were calculated with respect to H1 atoms of the glycosylating residues. The obtained results indicated that in the SASA approximation there should be a slight NOE from H-1 of the Fuc residue onto H-2 and H-3 of the GlcA unit. For non-sulfated compounds **1** and **4**, 2D-NOESY spectra were recorded and they showed no corresponding cross-peaks (Table 2).

**Table 2:** Relative experimental and calculated at SASA and explicit water level NOE values for structures **1** and **4**.

Compound Number	H1_(Fuc)_–H3_(GlA)_	H1_(Fuc)_–H4_(GlA)_	H1_(Fuc)_–H2_(GlA)_	H1_(Fuc`)_–H3_(Fuc)_	H1_(Fuc`)_–H4_(Fuc)_
**1**	**1 (exp)**	**0 (exp)**	**0 (exp)**	n.a.*	n.a.*
	1 (SASA)	0.26 (SASA)	0.12 (SASA)		
	1 (water)	0.05 (water)	0.01 (water)		
**4**	**1 (exp)**	**0 (exp)**	**0 (exp)**	**0.62 (exp)**	**0.7 (exp)**
	1 (SASA)	0.33 (SASA)	0.09 (SASA)	0.81 (SASA)	3.0 (SASA)
	1 (water)	0 (water)	0.01 (water)	0.7 (water)	1 (water)

* n.a.—Not applicable.

## 3. Experimental Section

### 3.1. Materials

The synthesis and detailed characterization of compounds **1**–**5** are described in [16]. For all NMR experiments, D_2_O was used as a solvent (99.98% D, Merck, Darmstadt, Germany). Samples for NMR spectroscopy were dissolved in D_2_O, lyophilized, dissolved in D_2_O again, and then transferred to NMR tubes for analysis.

### 3.2. Methods

All molecular mechanics calculation were done using TINKER v. 5.1 software package [21] with the implemented MM3 force field. The detailed description of MD simulations is provided in Section 2. For explicit water simulations a structure in question was soaked into a droplet of 2500 TIP3P [22] water molecules with automatic elimination of stacking molecules. The system was then equilibrated for 1 ns and additional 20 ns simulation was run with the snapshots being written every 2 ps. All hydrogen-involving bonds were constrained using RATTLE version of SHAKE [23] algorithm and cutoff value of 5 Å was applied.

The NMR spectra of **1**–**5** (10–20 mg) were recorded in D_2_O solutions on Bruker spectrometers AV-400 and AV-600 with 0.05% acetone as reference (^1^H 2.225 ppm, ^13^C 31.45 ppm) at the temperature of 303 K. Shigemi microtubes (purchased from Sigma-Aldrich, Inc., St. Louis, MO, USA) were sometimes used for sensitivity enhancement. The resonance assignment in ^1^H and ^13^C NMR spectra was performed by gradient enhanced 2D gCOSY, gNOESY, gHSQC, gJ-HMBC experiments as well as TOCSY and ROESY experiments.

NOE build-up was studied employing selective gradient enhanced 1D NOESY experiment with different mixing times. Other experimental NOEs were measured using a field gradient enhanced 2D gNOESY technique in D_2_O solutions at 303 K, mixing time 500 ms, relaxation delay 2 s. A sinusoidal field gradient of 1 ms length and a recovery time of 1 ms were used. The processing was performed with p/2 shifted sine-square function in both dimensions.

Experimental ^3^*J*_C–H_ constants were measured using J-HMBC [24] experiment. The spectral widths were about 2 ppm for ^1^H region and 40 ppm for ^13^C region and did not include resonances of aglycon groups. The data were collected in the echo/anti-echo mode. The length of gradients was 1 ms, and the recovery time was 100 us. The spectra were acquired with 80–120 t_1_ increments and 64–256 scans per increment. 1024 points were collected during the acquisition time t_2_. The HMBC preparation delay was set to 250 ms that corresponded to *J*_C–H_^min^ = 2.0 Hz. The upscaling coefficient k was 25–60. The relaxation delay was 1s. The third order low-pass J-filter was introduced for the suppression of one bond constant (^1^*J*_C–H_) in the range from 125 to 180 Hz. The forward linear prediction to 1024 points was used in F1. The processing was performed with p/2 shifted sine square function in both dimensions.

## 4. Conclusions

Five non-sulfated and selectively sulfated oligosaccharides of definite structure representing Fuc–GlcA and Fuc–Fuc–GlcA fragments of the side chains of fucosylated chondroitin sulfates (FCS) were studied by means of theoretical molecular mechanics calculations and NMR experiments. Long-range C–H coupling constants were used for the verification of the theoretical models. The presence of two conformers for both linkages was revealed. For the Fuc–GlA linkage, the predominant conformer was the same as described by other authors [11] as the MD average in a dodechasaccharide FCS fragment representing the backbone chain of the polysaccharide including GalNAc residues. Apparently, the introduction of a GalNAc substituent slightly impacts the conformation of the Fuc–GlA branch, just reducing the population of the second conformer. This shows that the studied oligosaccharides, in addition to larger ones, may be considered as reliable models for further QSAR studies in order to reveal pharmacophore fragments of FCS and particularly to understand the role of sulfate groups.
